# Myostatin promotes tenogenic differentiation of C2C12 myoblast cells through Smad3

**DOI:** 10.1002/2211-5463.12200

**Published:** 2017-02-20

**Authors:** Kazutaka Uemura, Masanori Hayashi, Toshiro Itsubo, Ayumu Oishi, Hiroko Iwakawa, Masatoshi Komatsu, Shigeharu Uchiyama, Hiroyuki Kato

**Affiliations:** ^1^ Department of Orthopaedic SurgeryShinshu University School of MedicineMatsumotoJapan; ^2^ Sports Medicine CenterAizawa HospitalMatsumotoJapan

**Keywords:** C2C12, myostatin, Smad3, tenocyte, tenomodulin

## Abstract

Myostatin, a member of the transforming growth factor‐β (TGF‐β) superfamily, is expressed in developing and adult skeletal muscle and negatively regulates skeletal muscle growth. Recently, myostatin has been found to be expressed in tendons and increases tendon fibroblast proliferation and the expression of tenocyte markers. C2C12 is a mouse myoblast cell line, which has the ability to transdifferentiate into osteoblast and adipocyte lineages. We hypothesized that myostatin is capable of inducing tenogenic differentiation of C2C12 cells. We found that the expression of scleraxis, a tendon progenitor cell marker, is much higher in C2C12 than in the multipotent mouse mesenchymal fibroblast cell line C3H10T1/2. In comparison with other growth factors, myostatin significantly up‐regulated the expression of the tenogenic marker in C2C12 cells under serum‐free culture conditions. Immunohistochemistry showed that myostatin inhibited myotube formation and promoted the formation of spindle‐shaped cells expressing tenomodulin. We examined signaling pathways essential for tenogenic differentiation to clarify the mechanism of myostatin‐induced differentiation of C2C12 into tenocytes. The expression of tenomodulin was significantly suppressed by treatment with the ALK inhibitor SB341542, in contrast to p38MAPK (SB203580) and MEK1 (PD98059) inhibitors. RNAi silencing of Smad3 significantly suppressed myostatin‐induced tenomodulin expression. These results indicate that myostatin has a potential role in the induction of tenogenic differentiation of C2C12 cells, which have tendon progenitor cell characteristics, through activation of Smad3‐mediated signaling.

**Abbreviations**
ActRIIB: activin receptor IIB
ALK: activin‐like kinase
FBS: fetal bovine serum
GDF: growth and differentiation factor
MAPK: mitogen‐activated protein kinase
MEK1: MAPK/Erk kinase 1
TGF‐β: transforming growth factor‐β
TSPCs: tendon‐derived stem/progenitor cells

Tendons play a critical role in the musculoskeletal system by transferring muscular forces to bone, thereby enabling joint movement. Treatment of tendon ruptures following injury is a clinical challenge for surgeons due to its hypocellular and hypovascular nature, which is exacerbated by the particularly limited knowledge of tendon biology. Therefore, a detailed understanding of molecular regulation of tendon differentiation may lead to the establishment of new strategies for tendon repair.

Recently, certain transcription factors essential for tendon differentiation have been identified. Scleraxis, a basic helix‐loop‐helix transcription factor has been reported to be a marker of tendon that is highly expressed in tendon progenitor cells 1, 2. Scleraxis positively regulates tenomodulin expression during tenocyte differentiation 3. The homeodomain protein mohawk is another transcription factor that has been identified to be an important regulator of tendon development 4, 5, 6. More recently, it has been reported that mice lacking the zinc finger transcription factor, early growth response‐1 (EGR1), displayed deficient tendon formation and expression of tendon genes, indicating the critical role of EGR1 in tendon development 7, 8.

The transforming growth factor‐β (TGF‐β) superfamily of growth factors is an important regulator of the differentiation of various types of cells and tissues 9. Some members of the TGF‐β superfamily of growth factors have also been shown to play essential roles in tendon formation. TGF‐β has been found to induce the expression of scleraxis and type I collagen *in vivo* 10, 11. Growth and differentiation factors (GDFs) have also been reported to induce tendon healing and differentiation of mesenchymal progenitor cells into tenocytes. GDF‐5 induces the tenogenic differentiation of adipose‐derived mesenchymal stem cells 12. GDF‐5 deficiency in mice leads to a delay in the Achilles tendon repair process 13. GDF‐6 deficiency in young male mice is associated with a substantial reduction in tendon total collagen 14. GDF‐7 gene transfer into a lacerated tendon improved tendon healing 15. GDF‐7 deficiency has a subtle effect on the composition and ultrastructure of murine Achilles tendon 16. Myostatin (GDF‐8) was originally identified as a negative regulator of muscle growth 17. Myoblast differentiation is negatively regulated by certain transcription factors and signaling pathways. Signaling pathways activated by myostatin have been reported, and are divided into Smad‐mediated and non‐Smad pathways 18. In the Smad‐mediated pathway, myostatin binds activin receptor IIB (ActRIIB) and Smad2/3 is phosphorylated via activin‐like kinase (ALK)‐4/5 19, 20. Philip *et al*. have reported that myostatin activates p38 mitogen‐activated protein kinase (MAPK) through TGF‐β‐activated kinase 1 (TAK1), and this appeared to be independent of Smad signaling 21. Yang *et al*. 22 demonstrated that myostatin activates Erk1/2 MAPK both in proliferating and differentiating C2C12 cells. Recently, Mendias *et al*. reported that deletion of myostatin in mice results in small, brittle, and hypocellular tendons, suggesting that myostatin regulates the structure and function of tendon tissue 23. Myostatin has also been shown to play a positive role in tendon maintenance and repair 24. Furthermore, the treatment of tendon fibroblast with myostatin increased cell proliferation and the expression of tenocyte markers, including scleraxis and tenomodulin 23. Meanwhile, in clinical medicine, several myostatin‐targeting drugs and treatment approaches for treating muscle atrophy have been developed. However, most of these do not consistently increase muscle size and function, and are fraught with off‐target problems. Moreover, although new treatment approaches for muscle atrophy have been reported to reduce the risk of off‐target effects 25, 26, effects on tendon have not been investigated and might serve to create weakened tendons and increase the risk for tendon injury. In this context, it is important to clarify the effect of myostatin on tenocyte differentiation.

The mouse myoblast cell line C2C12 was established from muscle satellite cells by Yaffe *et al*. 27. C2C12 is a multipotent progenitor cell line that has the ability to transdifferentiate into osteoblast and adipocyte lineages 28, 29. A previous study showed that C2C12 cells constitutively express scleraxis 30, suggesting that C2C12 is able to act as a tendon progenitor cell. Recently, Ker *et al*. 31 reported that bioprinting of fibroblast growth factor 2 (FGF2) onto aligned submicron fibrous scaffolds promotes transdifferentiation of C2C12 into tenocytes. Furthermore, Sassoon *et al*. 32 compared muscle‐derived stromal cells (MDSCs) with bone marrow stromal cells (BMSCs) and found that MDSCs have a propensity for tenocytic differentiation following GDF‐5 stimulation.

Based on these previous works, we hypothesized that myostatin can induce differentiation of C2C12 into a tenogenic lineage and negatively regulate myogenesis. We used a serum‐free culture system to examine the effect of myostatin on the differentiation of C2C12 into tenocytes. Furthermore, we investigated potent signaling pathways activated by myostatin to promote tenocyte differentiation. Our studies demonstrated that myostatin induces tenogenic differentiation of C2C12 cells through Smad3 signaling.

## Results

### C2C12 cells exhibit tendon progenitor cell characteristics

The myoblast cell line C2C12 and mesenchymal cell line C3H10T1/2 are both known to exhibit pluripotency. To examine the presence or absence of tendon progenitor cell characteristics in C2C12 and C3H10T1/2 cells, we compared the baseline expression level of scleraxis as a tendon progenitor cell marker. As expected, scleraxis expression was eightfold (7.90 ± 1.98) higher in C2C12 than in C3H10T1/2 cells. This result indicated that C2C12 cells have a characteristic of tendon progenitor cells, suggesting that they possess the potential to differentiate into tenocytes.

### Myostatin promotes commitment of C2C12 cells to the tenogenic lineage

**Figure 1:**
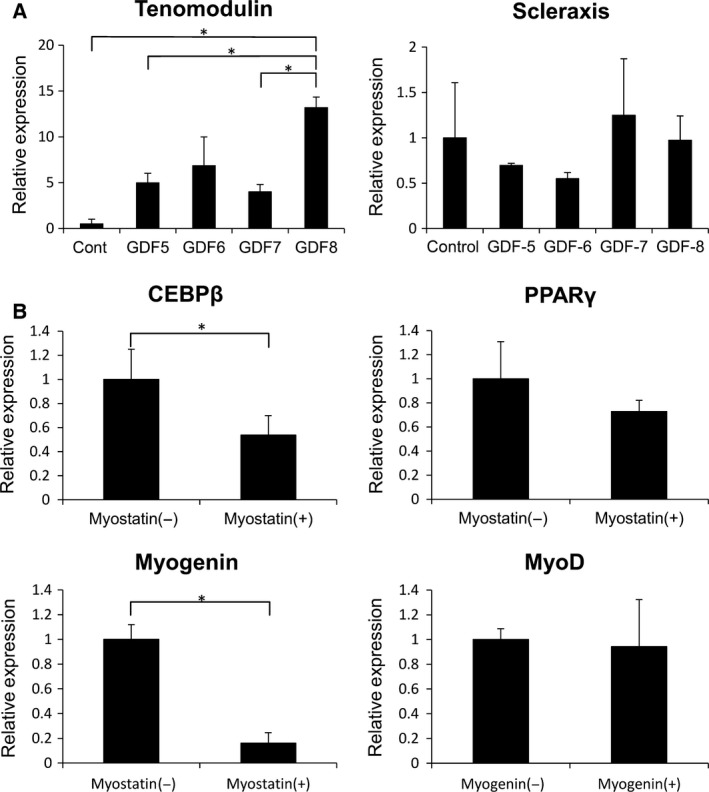
Myostatin treatment induces tenogenic differentiation and inhibits adipogenic and myogenic differentiation. (A) Relative expression of the tenogenic markers tenomodulin and scleraxis in C2C12 cells treated with GDF‐5, ‐6, ‐7, and ‐8 (myostatin) for 5 days. (B) Relative expression of adipogenic markers (CEBPβ and PPARγ) and myogenic markers (myogenin and MyoD) in C2C12 cells treated with or without myostatin for 5 days. Values represent the mean relative expression ± SEM. *N* = 4. *Statistically significant, *P* < 0.05.

Growth and differentiation factors have been reported to induce tenogenic differentiation of undifferentiated mesenchymal cells 12, 14, 15, 23. To determine which GDF induces differentiation of C2C12 into tenogenic lineage cells, C2C12 cells were treated with GDFs and the expression of tenogenic markers were quantitated. The expression of tenomodulin increased in the cells treated with all GDFs at day 5. In particular, myostatin induced the highest tenomodulin expression at 14‐fold (14.45 ± 0.58) higher than control. On the other hand, there was no remarkable change in the expression of scleraxis under these treatment conditions (Fig. 1A).

While myostatin inhibits myogenesis in C2C12 cells, it promotes differentiation of C3H10T1/2 into the adipogenic lineage 33. To determine the effect of myostatin on the differentiation of C2C12 cells into other mesenchymal cell lineages, myogenic and adipogenic markers were examined. The expression of the myogenic marker myogenin was suppressed from 1.02 ± 0.11 to 0.16 ± 0.09, and the adipogenic marker PPARγ was suppressed from 1.00 ± 0.08 to 0.73 ± 0.11 (Fig. 1B). These results suggest that myostatin promotes tenogenic differentiation as well as inhibits myogenic and adipogenic differentiation of C2C12 cells.

### Myostatin promotes proliferation and tenogenic differentiation of C2C12 cells in a time‐ and dose‐dependent manner

**Figure 2:**
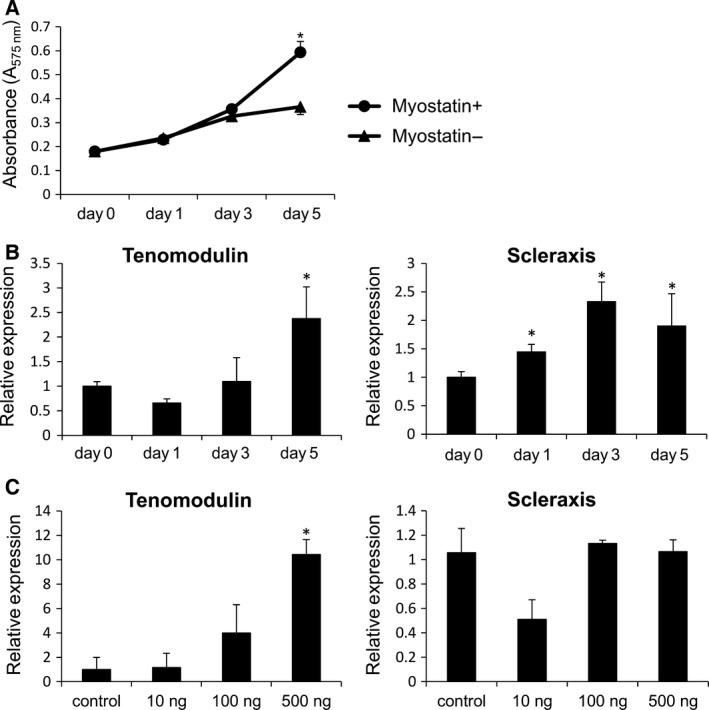
Myostatin promotes proliferation and tenogenic differentiation of C2C12 cells in a time‐ and dose‐dependent manner. (A) Cell proliferation of C2C12 cells cultured with or without myostatin was measured using the 3‐(4,5‐di‐methylthiazol‐2‐yl)‐2,5‐diphenyltetrazolium bromide (MTT) assay at days 0, 1, 3, and 5. (B) Tenomodulin and scleraxis expression was examined at 1, 3, and 5 days after myostatin stimulation. (C) C2C12 cells were treated with 10, 100, and 500 ng·mL^−1^ of myostatin or without myostatin (control) for 5 days. Relative expression of tenomodulin and scleraxis was assessed by real‐time PCR. Values represent the mean relative expression ±SEM. *N* = 4. *Statistically significant, *P* < 0.05.

Previous studies reported that recombinant myostatin generated in bacteria negatively regulates C2C12 proliferation 22. Recently, Rodgers *et al*. 34 demonstrated that recombinant myostatin generated in eukaryotic systems stimulates C2C12 proliferation. Therefore, we examined the effect of myostatin generated in eukaryotic systems on proliferation of C2C12 in our culture system. The proliferation of C2C12 cells with myostatin stimulation (0.593 ± 0.045) was increased at day 5 of cell culture compared to the C2C12 cells without myostatin stimulation (0.365 ± 0.032) (Fig. 2A). We quantified tenomodulin expression over time to examine changes of tenogenic marker expression after myostatin treatment. The expression of tenomodulin on day 5 was 2.4‐fold (2.38 ± 0.64) higher than on day 0. In contrast, scleraxis expression was the highest at 3 days after myostatin treatment. The expression of scleraxis on day 3 was 2.3‐fold (2.33 ± 0.57) higher than on day 0 (Fig. 2B). We also examined the effect of alteration in the dose of myostatin. The expression of tenomodulin was proportional to the dose of myostatin. The expression of tenomodulin in the presence of 500 ng·mL^−1^ of myostatin was 10‐fold (10.43 ± 1.22) higher than control. However, there was no significant change in scleraxis expression under these conditions (Fig. 2C). These findings suggest that myostatin promotes the proliferation and tenogenic differentiation of C2C12 in a time‐ and dose‐dependent manner.

### Myostatin induces tenomodulin expression in tenocyte‐like spindle cells

**Figure 3:**
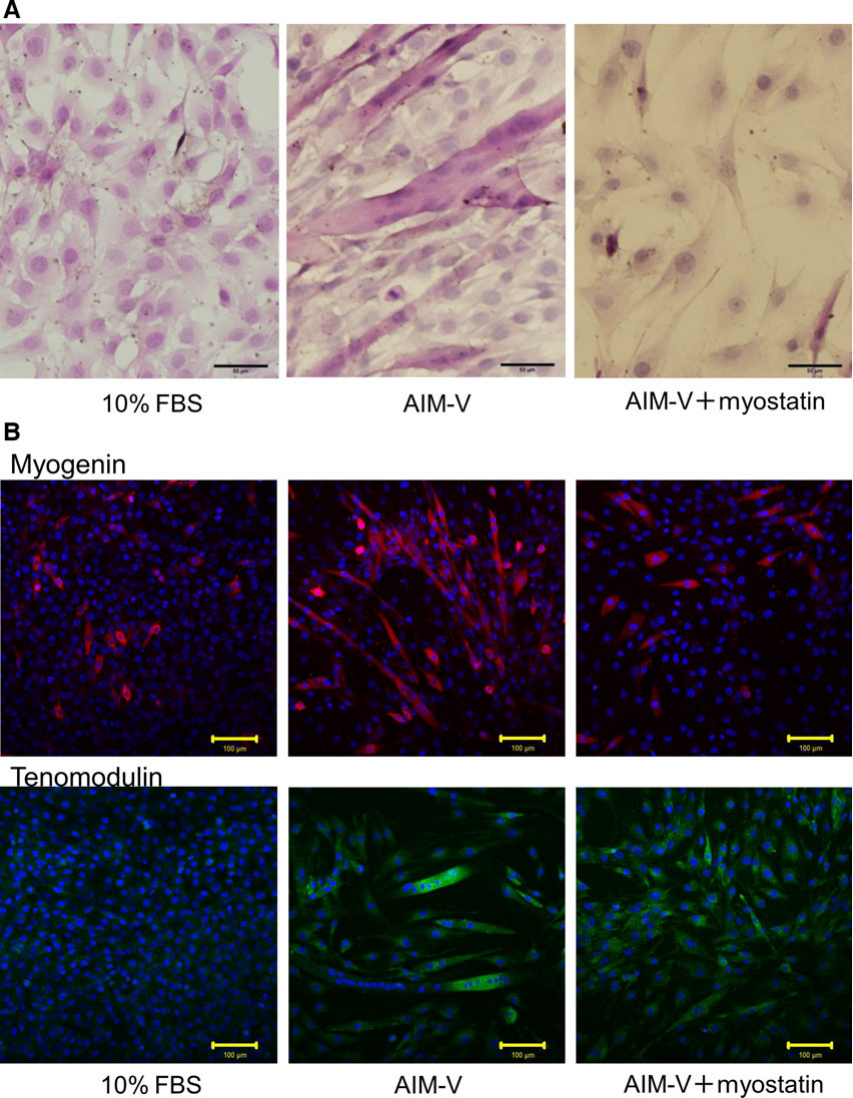
Myostatin induces tenomodulin expression in tenocyte‐like spindle cells. (A) Effect of myostatin on C2C12 cells was evaluated by Hematoxylin–Eosin (H‐E) staining. H‐E staining was performed after 5 days of culture in DMEM supplemented with 10% FBS, serum‐free AIM‐V medium with or without 500 ng·mL^−1^ of myostatin. Scale bar (black), 50 μm. (B) Immunofluorescence staining of myostatin and tenomodulin was performed after 5 days of cell culture. Green color indicates tenomodulin staining. Red color indicates myogenin staining. Nuclei were visualized by DAPI staining. Scale bar (yellow), 100 μm.

C2C12 cells form myotubes when cultured in low serum conditions 35. We examined the morphology of C2C12 cells after treatment with myostatin in serum‐free culture medium. While C2C12 cells cultured without myostatin formed myotubes, spindle‐shaped cells with a tenocyte‐like appearance were observed following myostatin treatment (Fig. 3A). Immunofluorescence showed that C2C12 cells expressed myogenin when cultured in serum‐free medium. A proportion of the cells also expressed tenomodulin. In contrast, C2C12 cells treated with myostatin did not form myotubes, and myogenin expression was suppressed. Tenomodulin was highly expressed in almost all of these cells (Fig. 3B).

### Myostatin induces tenogenic differentiation of C2C12 cells through the Smad2/3 signaling pathway

**Figure 4:**
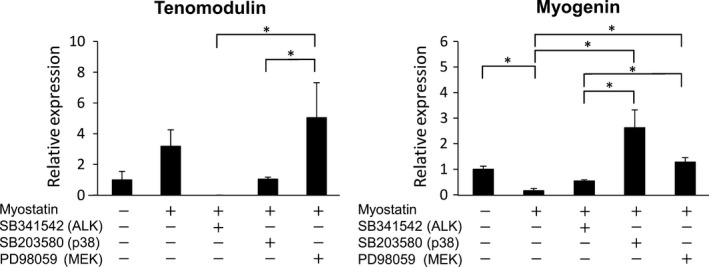
Myostatin induces tenogenic differentiation of C2C12 cells through the Smad2/3 signaling pathway. Relative tenomodulin and myogenin expression in C2C12 cells treated with myostatin and signaling pathway inhibitors. Cultured C2C12 cells were treated with or without 500 ng·mL^−1^ of myostatin in the presence or absence of 10 μmol·L^−1^ of inhibitors of ALK (SB341542), p 38 MAPK (SB203580), or MEK (PD 98059) for 5 days. Values represent the mean relative expression ±SEM. *N* = 4. *Statistically significant, *P* < 0.05.

Myostatin has been reported to activate certain signaling pathways, including ALK, p38MAPK, and Erk1/2 MAPK, after binding to ActRIIB 19, 20, 21, 22. In order to determine which signaling pathways are involved in myostatin‐induced tenogenic differentiation of C2C12 cells, inhibitors for each pathway were used during tenogenic induction. Tenomodulin expression was suppressed in C2C12 cells treated with myostatin and the ALK inhibitor SB341542 (0.0 ± 0.0). In the presence of p38MAPK (SB203580) and MEK1 (PD98059) inhibitors, the expression of tenomodulin was not significantly affected following myostatin treatment. The expression of tenomodulin treated with p38MAPK and MEK1 inhibitors was 1.04 ± 0.13 and 5.04 ± 2.3, respectively. Myostatin‐induced suppression of myogenin expression was rescued by treatment with all inhibitors (Fig. 4). These data suggest that myostatin induces tenogenic differentiation of C2C12 through the Smad2/3 signaling pathway and attenuates myogenesis via Smad2/3 and MAPK signaling.

### Smad3 is required for myostatin‐induced tenogenic differentiation of C2C12 cells

**Figure 5:**
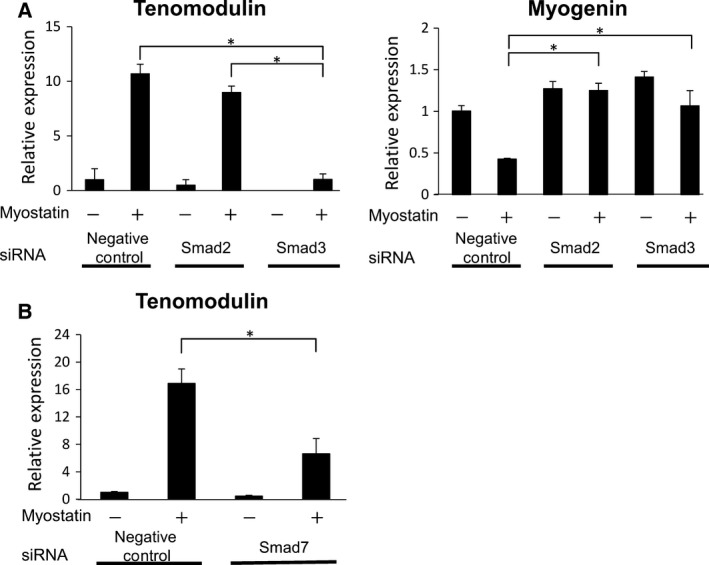
Smad3 is required for myostatin‐induced tenogenic differentiation of C2C12 cells. The effect of siRNA knockdown of Smad2 and 3 on myostatin signaling was assessed by RT‐PCR and immunofluorescence staining. siRNA (100 μm) targeting Smad2 (si‐Smad2), Smad3 (si‐Smad3), or nontargeting siRNA (negative control) was transfected into C2C12 cells. (A) Relative tenomodulin and myogenin expression in C2C12 cells treated with myostatin for 5 days after transfection of Smad2, Smad3, and negative control siRNA was assessed. (B) Relative tenomodulin expression in C2C12 cells treated with myostatin for 5 days after transfection of Smad7 and negative control siRNA was assessed. Values represent the mean relative expression ±SEM. *N* = 3. *Statistically significant, *P* < 0.05.

Protein down‐regulation with siRNA was used to examine the precise roles of the signaling factors Smad2 and Smad3 during tenogenic differentiation. When Smad2 was down‐regulated, no significant changes were observed in tenomodulin expression (from 10.68 ± 0.87 to 8.99 ± 0.55). Smad3 siRNA suppressed tenomodulin expression in C2C12 cells treated with myostatin (1.02 ± 0.51). Myostatin‐induced suppression of myogenin expression was rescued by treatment with Smad2 and Smad3 siRNA (Fig. 5A). These data suggest Smad3 is required for myostatin‐induced tenogenic differentiation of C2C12 cells. Smad7 was originally identified as an inhibitor of Smad2/3 signaling 36. Recently, Winbanks *et al*. 26 reported that smad7 gene delivery prevents muscle wasting of skeletal muscle. Therefore, we examined the effect of siRNA down‐regulation of smad7 on tenomodulin expression. This analysis showed that the expression of tenomodulin was unexpectedly suppressed by smad7 siRNA treatment with myostatin (Fig. 5B).

## Discussion

The current study is, to the best of our knowledge, the first to investigate the effect of myostatin on tenogenic differentiation of the C2C12 myoblast cell line. Compared to other GDFs, myostatin significantly up‐regulated the expression of a tenogenic marker under a serum‐free culture condition. In this study, the use of doses based on ng·mL^−1^, rather than absolute concentrations, is valid due to the comparable molecular weights of the various hormones. Myostatin was originally identified as a highly conserved TGF‐β family member that is expressed in developing and adult skeletal muscle 17. Deletion of myostatin in mice leads to a large and widespread increase in skeletal muscle mass, resulting from a combination of muscle cell hyperplasia and hypertrophy 17, suggesting that myostatin is a negative regulator of skeletal muscle growth. However, the effect of myostatin on muscle‐derived cells varies according to the cell type. Myostatin inhibits satellite cell proliferation and activates differentiation 22, 37, 38, 39, 40, 41. Meanwhile, myostatin inhibits C2C12 differentiation into myotubes 22. Although there are many reports investigating the behavior of myostatin in myogenesis, none of the previous studies have investigated the cell lineages in which myostatin promotes differentiation of C2C12 myoblasts.

Myostatin is initially expressed in both myogenic and nonmyogenic cells at the early stage of limb bud development and is localized to many different muscles during the late limb bud stage 17, 42. The expression pattern of myostatin is heterogeneous, even within each specific muscle 42. The expression of myostatin has also been detected in adult tendon, and plays a positive role in tendon maintenance and early repair 23, 24, 43, 44. In our culture model, myostatin increased the expression of a tenogenic marker and inhibited a myogenic marker in C2C12 cells. The effect of recombinant myostatin on the proliferation of C2C12 has been controversial 22, 34. In our study, we used recombinant myostatin generated in eukaryotic systems and confirmed the positive effect of myostatin on C2C12 proliferation, as reported by Rodgers *et al*. 34. These results indicate that myostatin promotes tenogenic differentiation of myoblasts as well as inhibits myogenesis during muscle development. Recently Bi *et al*. 45 reported that human and mouse tendon contains a minor cell population with stem cell properties, named tendon‐derived stem/progenitor cells (TSPCs). TSPCs express higher levels of scleraxis compared to bone marrow stromal cells (BMSCs) and regenerate tendon‐like tissue. Mendias *et al*. 23 reported that treatment of fibroblasts isolated from tendon with myostatin increases the expression of tenogenic markers, including scleraxis and tenomodulin. Meanwhile, the expression of scleraxis in C2C12 cells has been previously reported 30. In the current study, we also detected scleraxis expression in C2C12 cells. Given that C2C12 cells are derived from myoblasts in mouse muscle 27, these results strongly suggest that myoblasts share characteristics in common with TSPCs, in that both cells have the potential to act as tendon progenitor cells. Therefore, it is quite possible that myostatin promotes differentiation of TSPCs into tenocytes as it does in C2C12 cells.

It has been reported that myostatin stimulates muscle fibroblast proliferation and induces differentiation into myofibroblasts 46, 47. Myostatin has been shown to be expressed in myogenic and nonmyogenic cells during muscle regeneration and in C2C12 cells 47, 48. Expression of activin receptor IIB (ActRIIB), a putative receptor for myostatin, has also been detected in muscle fibroblasts and C2C12 cells 47. Although the expression of scleraxis in muscle fibroblasts that express the fibroblast markers vimentin, heat shock protein 47 (HSP47), and α‐smooth muscle actin (α‐SMA) was not determined, the induction of tenogenic differentiation by myostatin may depend on whether or not the cells express scleraxis. The effects of myostatin on adipogenic differentiation have also been reported; however, the results remain controversial. Artaza *et al*. 33 reported that myostatin promotes the differentiation of the multipotent mesenchymal cell line C3H10T1/2 into an adipogenic lineage. Although other studies have shown that myostatin inhibits adipogenic differentiation of 3T3‐L1 preadipocytes and bone morphogenetic protein 7 (BMP7)‐induced adipogenic differentiation of C3H10T1/2 cells 20, 49. These discrepancies could be caused by the degree of cell differentiation and culture conditions. In particular, cell differentiation status could have a much greater impact on the reaction of cells after stimulation by growth factors. Recently, Li *et al*. reported that the expression of adipogenic markers of the stromal vascular cellar fraction, which is analogous to the stem cell pool, transit amplifying and progenitor cells in adipose tissue, is elevated in myostatin null mice 50. In our culture model, the expression of adipogenic markers was suppressed by myostatin, suggesting that C2C12 cells have a similar characteristic as stromal vascular cells in adipose tissue.

We unexpectedly found that the expression of tenomodulin in myotubes was induced under serum‐free culture conditions. It has been reported that myostatin is expressed in myotubes but not in C2C12 myoblasts 51. This report suggests that endogenous myostatin in myotubes exerts an effect on the expression of tenomodulin through an autocrine mechanism. We have also observed the phenotypical heterogeneity of C2C12 cells stimulated by myostatin. The heterogeneity of Myf5 expression levels in C2C12 has been previously reported 52. Cell diversity in C2C12 upon serum deprivation has also been shown 53. The C2C12 cell line is comprised of varied populations of cells including CD34‐positive cells and cells with the side population (SP) phenotype 54, 55. These findings could partially explain the diverse response found in our culture model. Further studies identifying the fraction of cells with a tenogenic progenitor nature in C2C12 cultures may provide additional evidence for this heterogeneous response.

To gain insight into the molecular mechanism of the tenogenic differentiation of C2C12 cells induced by myostatin, we have examined the signaling pathway that is essential for tenogenic differentiation. Myostatin has been shown to elicit its function by binding to the ActRIIb and type Ib (ActRIB) receptors, followed by phosphorylation of common downstream effectors, including Smad2, Smad3, Erk1/2, and p38 MAP kinase 20, 21, 22. Our data showed that the expression of tenomodulin was significantly inhibited by treatment with a Smad2/3 inhibitor, suggesting that the activation of the Smad2/3 signaling pathway was essential for tenogenic differentiation of C2C12 cells. Furthermore, we used siRNA to selectively down‐regulate the expression of Smad2 and Smad3, and found that the activation of Smad3 contributed to tenogenic differentiation. Recently, Smad3 has been reported to bind scleraxis and Mohawk, and regulate normal tendon formation and maintenance of mature tendon 56. In the current study, we established that activation of Smad3 was required for tenogenic differentiation induced by myostatin. On the other hand, down‐regulation of Smad7 resulted in suppression of tenomodulin expression. This may be caused by a difference between the mechanism of inhibition of myogenesis and activation of tenogenesis by myostatin. Specifically, Smad2 is not involved in a regulation of tenocyte differentiation by myostatin, which is thought to be one of the reasons for the unexpected result. Moreover, the expression of tenomodulin in myotubes and the phenotypical heterogeneity of C2C12 cells may be the causes of this result.

Understanding of the mechanism of tenocyte differentiation still lags in comparison to other mesenchymal lineage cells. One of the major reasons for this is that a standard culture system using a cell line has not been well established. In the current study, we used the myoblastic cell line, C2C12, in combination with serum‐free culture medium to achieve a simpler and more reproducible experimental system. As a result, we successfully established a useful culture system for observing tenocyte differentiation. Furthermore, it has been reported that muscle‐derived stromal cells have the potential to repair tendon through differentiation into tenocytes 32, 57. Our data support these reports and expand the possibility of using myoblastic cells as an ideal source for tenocytic differentiation. Further investigation using our culture system will elucidate the molecular mechanism of tendon differentiation and may provide new therapeutic options for tendon injury.

## Materials and methods

### Cell culture and induction of differentiation

C2C12 and murine mesenchymal cells (C3H10T1/2) were cultured in Dulbecco's modified Eagles medium (DMEM) supplemented with 10% FBS and antibiotics. The cells were passaged before reaching confluence and used within 10 passages. To induce tenocyte differentiation, C2C12 cells were cultured in serum‐free AIM‐V medium (Thermo Fisher Scientific, Waltham, MA, USA) supplemented with GDFs (500 ng·mL^−1^ of GDF‐5, ‐6, ‐7 and 10, 100, 500 ng·mL^−1^ of myostatin; R&D Systems, Minneapolis, MN, USA). The medium was replaced every 2 days. To analyze signaling pathways, cells were treated with 500 ng·mL^−1^ of myostatin and 10 μmol·L^−1^ of inhibitors for ALK (SB431542), p38MAPK (SB203580), and MEK1 (PD98059) (Abcam, Cambridge, MA, USA) for 5 days.

### Real‐time PCR

Total RNA was isolated from cultured cells using an RNeasy Mini kit (Qiagen, Hilden, Germany). RNA was reverse‐transcribed to cDNA using the ThermoScript RT‐PCR System (Thermo Fisher Scientific). Quantitative real‐time PCR was performed with TaqMan Gene Expression Master Mix (Thermo Fisher Scientific) using a StepOnePlus real‐time PCR system (Thermo Fisher Scientific). TaqMan Gene Expression Assay Probes for scleraxis (Mm01205675_m1), myostatin (Mm00491594_m1), MyoD (Mm00440387_m1), myogenin (Mm00446195_g1), PPARγ (Mm01184322_m1), CEBPβ (Mm00843434_s1), Smad2 (Mm00487530_m1), Smad3 (Mm01170760_m1), and hypoxanthine phosphoribosyltransferase 1 (HPRT) (Mm00446968 m1) were purchased from Thermo Fisher Scientific. The relative mRNA expression levels of target genes were calculated as fold changes of the threshold cycle (Ct) value relative to a reference using the 2‐ΔΔCt method. HPRT was used as the reference gene.

### MTT assay

Quantification of cell proliferation and viability was performed using a Cell Proliferation Kit I (Roche, Basel, Switzerland). Briefly, C2C12 cells were seeded in microplates and cultured using medium supplemented with 500 ng·mL^−1^ of myostatin for 1–5 days. After the culture period, 10 mL of the 3‐(4,5‐di‐methylthiazol‐2‐yl)‐2,5‐diphenyltetrazolium bromide (MTT) labeling reagent was added to each well. The microplates were incubated at 37 °C in a 5% CO_2_ humidified incubator for 4 h. Into each well was added 100 mL of the solubilization solution. Samples were incubated at 37 °C in a 5% CO_2_ humidified incubator overnight. Absorbance (575 nm) was measured using a VERSA Max Plus (Molecular Devices, Sunnyvale, CA, USA).

### Immunofluorescence

Cells were fixed with 4% paraformaldehyde in PBS 5 days after GDF‐8 treatment. Fixed cells were blocked in PBS‐T containing 2% BSA for 1 h, and then incubated overnight at 4 °C with anti‐tenomodulin (Santa Cruz Biotechnology, Dallas, TX, USA) and anti‐myogenin (Abcam) antibodies. Subsequently, cells were stained with Alexa Fluor 488‐ and 588‐conjugated goat anti‐(rabbit IgG) secondary antibody (Thermo Fisher Scientific) at room temperature for 1 h. Cells were then mounted using the Vectashield HardSet Mounting Medium with 4′,6‐diamidino‐2‐phenylindole (DAPI) (Vector Laboratories, Burlingame, CA, USA).

### RNA interference

Target short interfering RNA (siRNA) for Smad2 (Silencer Select Pre‐Designed siRNA s69492), Smad3 (s69494), and Smad7 (s69506) were purchased from Thermo Fisher Scientific. C2C12 cells were seeded at 30% confluence on the day before transfection. The siRNA transfections were performed using RNAi Max transfection reagent (Thermo Fisher Scientific). The effects of target‐specific siRNA were confirmed by real‐time PCR for Smad2 and 3 at 24 h and 5 days after siRNA transfection. At 1 h after siRNA transfection, cells were treated with myostatin (500 ng·mL^−1^) for 5 days.

### Statistical analysis

The data are presented as means ± SEM. Comparisons were made using a *t* test to determine the significance of differences between two groups. For multiple comparisons, differences between means were determined by a one‐way ANOVA coupled with Dunnet's, Williams’ or Tukey's *post hoc* tests. In each comparison, *P* < 0.05 was set as the level of significance.

## Author contributions

MH and TI planned experiments. KU and AO performed experiments. KU, AO, HI, and MK analyzed data. KU and MH wrote the paper; and TI, SU, and HK reviewed the manuscript.
